# The clinical application value of mixed reality in robotic laparoscopic partial nephrectomy

**DOI:** 10.3389/fonc.2024.1478051

**Published:** 2024-11-06

**Authors:** Xin Chang Zou, Xiang Da Xu, Jian Biao Huang, Hai Chao Chao, Tao Zeng

**Affiliations:** ^1^ The Second Affiliated Hospital, Jiangxi Medical College, Nanchang University, Nanchang, China; ^2^ Department of Urology, Second Affiliated Hospital of Nanchang University, Nanchang, China

**Keywords:** mixed reality, renal cell carcinoma, R.E.N.A.L score, enhanced CT, robotic-assisted laparoscopic partial nephrectomy

## Abstract

**Purpose:**

Robot-assisted laparoscopic partial nephrectomy (RAPN) has become a key technology in the treatment of renal tumors. Effective preoperative planning and precise intraoperative navigation are critical to a successful surgical outcome. This study aimed to evaluate the clinical application value of mixed reality (MR) in robotic nephron-sparing partial nephrectomy for patients with renal tumors of different complexity based on the R.E.N.A.L. score.

**Patients and methods:**

A retrospective analysis was conducted on 68 eligible patients with renal cancer who underwent RAPN at The Second Affiliated Hospital of Nanchang University from January 2021 to December 2023, with postoperative pathology confirmation. Patients were divided into two groups: the MR group, with 28 cases, and the traditional imaging (control) group, with 40 cases. All patients underwent mid-abdominal CT plain scans and enhancements. The MR group utilized three-dimensional reconstruction of CT data and employed 3D tablets and HoloLens glasses for preoperative discussions, surgical planning, and intraoperative guidance. Collect clinical data and metrics to assess surgical outcomes, as well as evaluate performance in areas such as preoperative discussions, doctor-patient communication, surgical planning, and intraoperative navigation.

**Results:**

Compared to robot-assisted partial nephrectomy in the control group, the MR group experienced a reduction in operation time by approximately 30 min [(135.89 ± 23.494) min vs. (165.00 ± 34.320) min, P< 0.001)] and a decrease in ischemia time by around 2.5 min [(20.36 ± 3.956) min vs. (23.80± 6.889) min, P = 0.02)]. Within the subgroup with a R.E.N.A.L. score of less than 7 points, the MR group only showed a significant reduction in operation time [(134.55 ± 150.190) min vs. (150.19 ± 28.638) min, P = 0.045], with no notable differences in other parameters. For the subgroup with a R.E.N.A.L. score of 7 points or higher, the MR group exhibited shorter operation time [(140.83 ± 25.183) min vs. (195.77 ± 23.080) min, P< 0.001] and reduced warm ischemia time [(21.17 ± 2.714) min vs. (28.85 ± 7.570) min, P = 0.029]. Additionally, there was less estimated blood loss [(53.33 ± 5.164) min vs. (114.62 ± 80.376) min, P = 0.018]. All patients had negative resection margins, indicating equivalent therapeutic outcomes between the two groups.

**Conclusion:**

In comparison to traditional RAPN, MR technology demonstrates the ability to decrease operation time and warm ischemia time all the while maintaining equivalent curative outcomes. Additionally, it enhances preoperative discussions, doctor-patient interactions, preoperative strategizing, and intraoperative navigation, particularly excelling in complex renal tumor cases of RAPN, where its benefits are most pronounced.

## Introduction

Renal cell carcinoma, constituting approximately 3% of all cancers, ranks third in incidence among urinary system tumors and bears the highest mortality rate within this category. The primary age of onset falls between 50 and 60 years, with a higher incidence rate in men compared to that in women (1, 2). The etiology of kidney cancer remains unclear, although smoking, obesity, and body mass index (BMI) (<25 or>35) are established risk factors (3, 4). Renal cancer predominantly arises from renal tubular epithelial cells, representing roughly 90% of renal cancers and is commonly referred to as renal cell carcinoma or renal adenocarcinoma (5).

Currently, surgical intervention remains the primary approach for managing early-stage localized renal cancer in clinical settings, with nephron-sparing partial nephrectomy representing the established standard treatment for T1 stage renal tumors. The evolution of minimally invasive techniques has transformed partial nephrectomy from its initial open surgery form to the prevailing laparoscopic partial nephrectomy and robot-assisted laparoscopic partial nephrectomy (RAPN) methods (6). Numerous studies and data have demonstrated that robotic procedures offer superior outcomes in terms of operation duration, blood loss, warm ischemia time, and other factors compared to laparoscopic surgery all while maintaining equivalent tumor outcomes and progression-free survival rates (7–9). In 2009, Kutikov et al. introduced the systematic and standardized R.E.N.A.L. score for renal tumors, incorporating factors such as the maximum tumor diameter (R), exophytic/endophytic tumor characteristics (E), tumor’s proximity to the collecting system or renal pelvis (N), distance from the tumor to the kidney’s ventral or dorsal aspect (A), and the tumor’s relation to the upper/lower pole of the kidney (L). This scoring system has proven effective in predicting surgical complexity and perioperative complication rates following extensive validation, aiding in surgical method selection (partial nephrectomy or radical nephrectomy) and outcome prognostication (10–12).

Mixed reality (MR) technology seamlessly blends the real world with virtual elements, representing a significant advancement beyond virtual reality and augmented reality. By facilitating interactions between the virtual and physical realms, it offers users a novel and immersive experience. Typically accessed through head-mounted devices, users can manipulate virtual objects to alter their environment and engage with them through sensor-based interactions. The concept of MR was initially introduced by Milgram and Kishino and has since found applications in diverse fields such as education, healthcare, architecture, and entertainment (13). In the medical domain, MR technology plays a crucial role in enhancing preoperative planning for procedures like partial nephrectomy by providing detailed three-dimensional (3D) visual reconstructions of tumors and renal anatomy. This capability not only improves preoperative discussions but also enhances surgical outcomes (14, 15).

This study integrates the R.E.N.A.L. score to investigate the clinical utility of MR in robotic nephron-sparing partial nephrectomy for renal tumors of different complexities.

## Patients and methods

### Patients

This retrospective study and its project were approved by the Biomedical Research Ethics Committee of The Second Affiliated Hospital of Nanchang University, and the requirement for patient informed consent was waived. Collect relevant data on 68 suitable patients with renal tumors who underwent nephron-sparing nephrectomy surgery at The Second Affiliated Hospital of Nanchang University from January 2021 to December 2023. These data should include patient age, gender, height, weight, BMI, presence of underlying diseases, maximum tumor diameter, R.E.N.A.L. score, presence of renal artery variations (such as accessory renal artery or premature branching), preoperative hemoglobin and blood creatinine levels, American Society (ASA) classification, surgery duration, renal warm ischemia time, postoperative hemoglobin and blood creatinine levels, estimated intraoperative bleeding volume, extubation time, postoperative hospital stay, postoperative complications (e.g., urinary fistula and collecting system injury), pathology type, surgical margins, and tumor stage.

**Figure 1:**
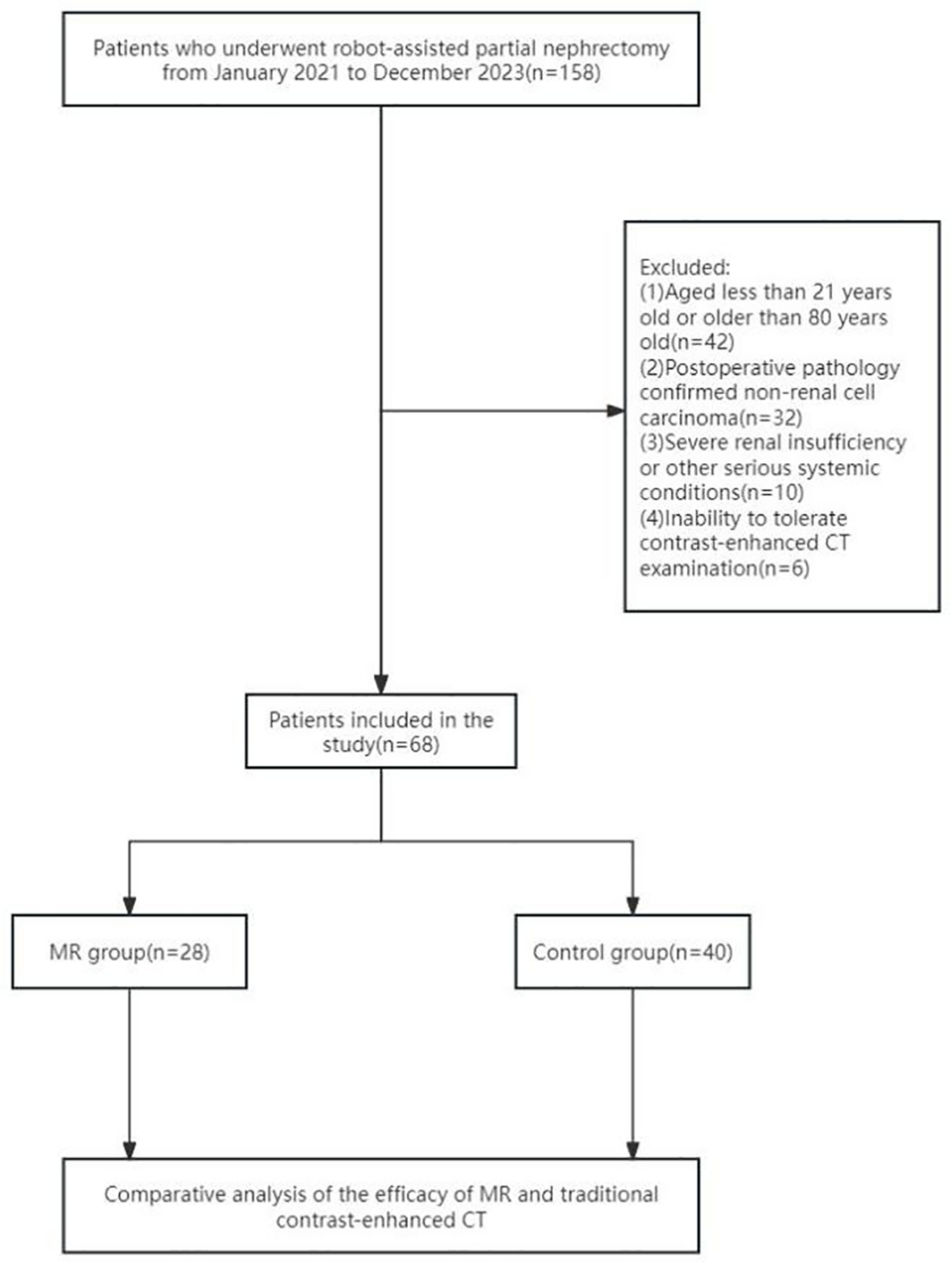
Flowchart for case screening.

All patients underwent preoperative CT scans of the mid-abdomen (including both kidneys) and pelvis with contrast enhancement. Inclusion criteria were as follows: (1) age between 21 and 80 years; (2) patients with renal tumors diagnosed as T1 stage following preoperative enhanced CT scans; (3) surgeries performed by experienced urological surgeons; and (4) postoperative pathology confirmed renal cell carcinoma. Exclusion criteria included the following: (1) severe renal insufficiency or having a single functional kidney; (2) allergy to CT contrast agents; (3) abnormal coagulation function or use of oral anticoagulants; (4) severe cardiovascular, cerebrovascular, or systemic diseases. The screening flowchart is shown in **Figure 1**.

The participants were grouped on the basis of the use of MR technology. The study included 28 patients in the MR group and 40 patients in the CT-enhanced group (who did not utilize MR technology). Both groups of patients had the same indications for RAPN. Additionally, patients were stratified based on a R.E.N.A.L. score of 7 as the threshold. Patients with a score equal to or greater than 7 (indicative of medium-high complexity renal tumors) were compared to those with a score below 7 (representing low-complexity renal tumors). Among the low-complexity renal tumor subgroup, there were 49 cases in total (22 in the MR group and 27 in the control group), whereas the highly complex renal tumor subgroup comprised 19 cases (6 in the MR group and 13 in the control group).

### Image data collection and mixed reality model reconstruction

**Figure 2:**
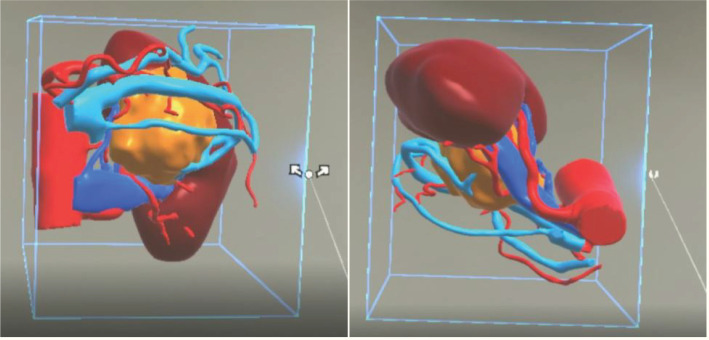
A three-dimensional virtual model generated from imported DICOM data based on Smart Vision SDVWorks software.

All patients underwent a plain scan followed by an enhanced CT scan of the mid-abdomen (both kidneys) and pelvis. To ensure consistency in the MR reconstruction model, patients were scanned using our hospital’s GE Light Speed 64-slice 128-slice VCT machine, with a slice thickness of 1.25 mm. Patients were positioned supine, and breathing was controlled as per guidance. Initially, a routine plain scan was conducted, followed by the injection of iohexol-enhanced contrast agent through the cubital vein. Subsequently, scans were taken during the arterial phase, venous phase, and delayed phase, respectively. The control group engaged in preoperative discussions and surgical planning based on the patients’ CT images and MultiPlanar (MPR) multidimensional images. The MR team exported the patient’s image data in DICOM format to medical image processing software (Smart Vision SDVWorks, Shenzhen Yitu Co., Ltd.), selecting thin-slice images (1.25mm thickness) corresponding to the various scan phases. Kidneys, tumors, collecting systems, lymph nodes, blood vessels, and other structures were segmented using specific modules. Any unidentified areas were manually segmented. The renal parenchyma and arteries were highlighted in red, veins in blue, and tumors in yellow. The resulting image was then viewed using HoloLens glasses and a Nubian naked-eye 3D tablet (refer to **Figure 2**).

### Mixed reality applications and surgical methods

MR group robot-assisted laparoscopic nephrectomy: Prior to the surgery, the patient’s CT data were reconstructed, and the resulting MR model was integrated into the Nubian naked-eye 3D tablet and HoloLens glasses (an MR head-mounted display by Microsoft Corporation). The Nubian naked-eye 3D tablet was utilized to interact with patients, providing information on tumor location, surgical procedures, associated risks, and complexities, thereby enhancing patients’ comprehension of the illness, treatment options, and medical personnel. Simultaneously, HoloLens glasses were employed to manipulate the visibility of various tissues and organs, adjust viewing angles, zoom in and out on specific areas and overall structures, modify distances, and segment components and the entirety, facilitating real-time monitoring of tumors, kidneys, surrounding blood supply, and the collection system. This enabled a comprehensive understanding of adjacent structures and their interconnections, aiding in the determination of surgical methods, approaches, and the estimation of the positions of blood vessels, tumors, and collection systems, ensuring meticulous preoperative planning.

The surgical procedure follows the transperitoneal approach. To begin, following anesthesia, the patient is positioned supine with the contralateral side exposed. After standard disinfection and draping, a 1.5-cm incision is made 2 cm lateral to the contralateral umbilicus. Pneumoperitoneum is then established using a pneumoperitoneum needle. A robot Trocar is inserted as the two-arm observation hole channel. Using this point as a reference, additional Trocar channels are created along the outer edge of the rectus abdominis on the affected side and at the iliac crest. A four-arm channel is established above, with an auxiliary channel positioned two transverse fingers above the iliac crest. The mechanical arm is then connected. The robot assists in examining the abdominal cavity, exposing the prerenal fascia by incising along the paracolic groove.

**Figure 3:**
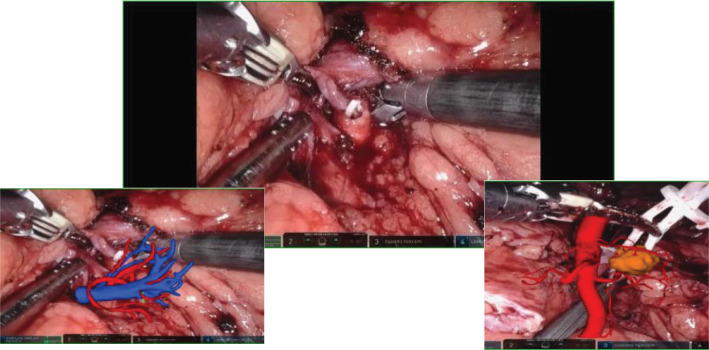
Intraoperative mixed reality models are superimposed onto live anatomy to improve visualization of the renal hilum and tumor vessels.

**Figure 4:**
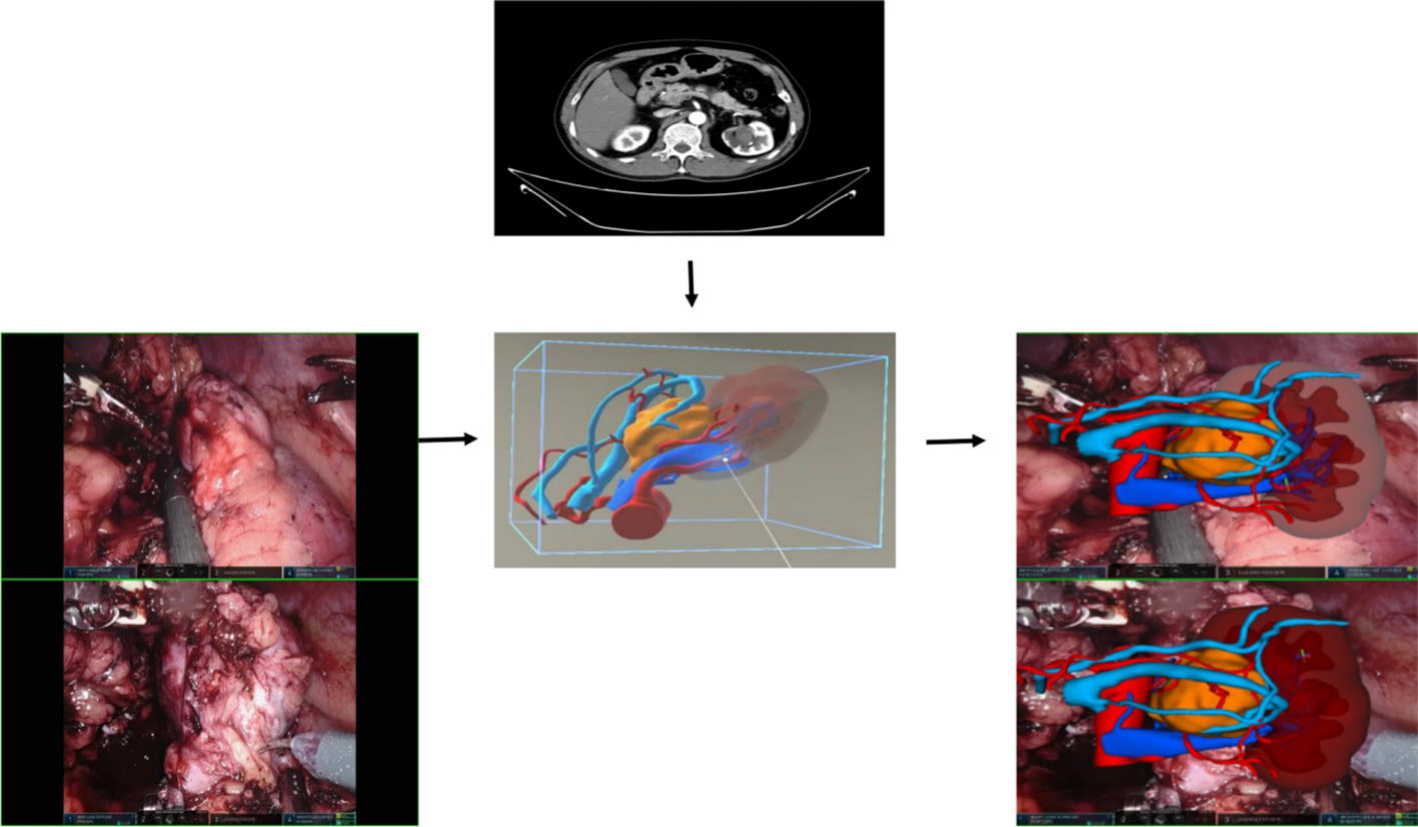
Mixed reality technology plays a crucial role in the process of kidney separation and accurate tumor location identification. Through advanced mixed reality models, blood vessels and tissues can be precisely identified and targeted during tumor separation and resection procedures.

**Figure 5:**
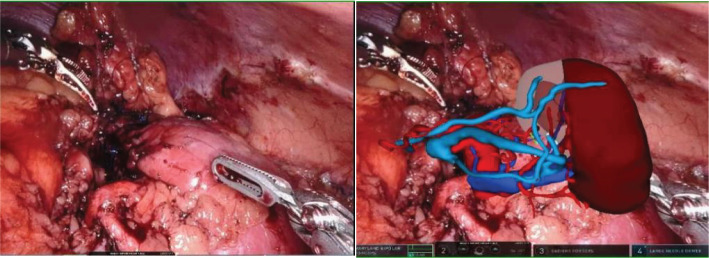
After the tumor is removed, the MR model is once again overlaid to clearly display the kidney tissue and blood vessels. This allows for a detailed examination of any suspicious bleeding points and resection margins post-surgery.

HoloLens glasses aid in identifying tumor location, surrounding blood vessels, and collecting systems (refer to **Figure 3** for visual representation). The tumor and renal arteries are fully exposed, blood supply to the kidney is blocked, and the tumor, along with a portion of renal parenchyma, is excised (**Figure 4**). Renal calyces are sutured at the wound base with 3-0 Vicryl interrupted sutures, and bleeding points are addressed with 2-0 continuous sutures along the renal incision (**Figure 5**). Once bleeding is controlled, the specimen is removed, a perinephric drain is placed through the axillary incision, and pneumoperitoneum is released. Each incision is sutured layer by layer using the robotic system. The procedure concludes with the restoration of blood supply to the kidney. For the retroperitoneal approach, tumor location, blood vessels, and collecting systems are estimated on the basis of the spine and costal margins.

### Statistical analysis

In this study, statistical analysis of data was conducted using SPSS 27.0 software. The measurement data were assessed for normality, with data conforming to a normal distribution presented as mean ± standard deviation. Independent sample T-tests were utilized. In cases where the data did not meet the criteria for normal distribution, median and quartiles were used, and the Mann–Whitney U-test was applied. Categorical variables were represented as numbers (%) and analyzed using the chi-square test. Bilateral P-value of <0.05 was considered statistically significant.

## Result

### Patients

There were no statistically significant differences in the preoperative data between the two groups of patients (refer to **Table 1** for details). Upon comparing the intraoperative and postoperative data between the two groups, it was evident that the MR group exhibited more favorable outcomes in terms of average operating time, average warm ischemia time, and estimated intraoperative blood loss in contrast to the control group (p< 0.05). However, no statistical variances were observed in other pertinent intraoperative and postoperative indicators. Please refer to **Table 2** for detailed information. In the MR group, there were no postoperative complications observed. All resection margins were negative. In the control group, three cases of postoperative complications occurred, including one case of collecting system injury and two cases of urinary fistula. All resection margins were negative.

**Table 1:** Comparison of preoperative general information.

			(%), $\bar{\text{x}}$ ± S, M (Q_1_, Q_3_)
	MR group (n = 28)	Control group (n = 40)	P
Age, mean ± sd (year)	58.96 ± 13.14	55.35 ± 12.42	0.253
Gender, n (%)			0.518
Male Female	19 (67.9%) 9 (32.1%)	30 (75.0%) 10 (25.0%)	
Height, mean ± sd (cm)	165.07 ± 8.67	165.75 ± 7.70	0.735
Weight, mean ± sd (kg)	66.25 ± 12.32	65 (57.75, 72.50)	0.817
BMI, mean ± sd (kg/m^2^)	24.24 ± 3.68	23.93 ± 3.39	0.728
R.E.N.A.L. score (Q_1_, Q_3_)	6 (5, 6)	6 (5, 7)	0.702
Tumor side, n (%)			0.451
Left Right	13 (46.4%) 15 (53.6%)	26 (65.0%) 14 (35.0%)	
Renal artery variation			0.861
No Yes	16 (57.1%) 12 (42.9%)	22 (55.0%) 18 (45.0%)	
Preoperative hemoglobin, mean ± sd (g/L)	140.96 ± 15.56	139.50 ± 14.25	0.689
Preoperative serum creatinine (Q_1_, Q_3_) (μmol/L)	76.50 (62.25, 96.75)	79.50 (64.00, 91.50)	0.727
ASA			0.429
1 2 3	1 (3.4%) 23 (82.1%) 4 (14.3%)	5 (12.5%) 29 (72.5%) 6 (15.0%)	

**Table 2:** Comparison of intraoperative and postoperative data.

			(%), $\bar{\text{x}}$ ± S, M (Q_1_, Q_3_)
	MR group (n = 28)	Control group (n = 40)	P
Operation time, mean ± sd (min)	135.89 ± 23.49	165.00 ± 34.33	<0.001
Warm ischemia time, mean ± sd (min)	20.36 ± 3.97	23.80 ± 6.899	0.020
Estimated amount of bleeding (Q_1_, Q_3_) (mL)	60 (50, 100)	90 (60, 110)	0.013
Postoperative hemoglobin, mean ± sd (g/L)	122.07 ± 15.84	119.68 ± 15.53	0.537
Postoperative serum creatinine (Q_1_, Q_3_) (µmol/L)	91.00 (80.00, 125.25)	93.00 (79.00, 115.25)	0.562
Hemoglobin changes (Q_1_, Q_3_) (g/L)	17.5 (10.25, 24.75)	19 (11.25, 26.00)	0.694
Changes in serum creatinine (Q_1_, Q_3_) (µmol/L)	13.00 (2.25, 16.75)	8.00 (4.25, 15.00)	0.798
Postoperative hospital stay (Q_1_, Q_3_) (day)	7 (6, 8)	7 (6, 8)	0.563
Postoperative extubation time (Q_1_, Q_3_) (day)	6 (5, 7)	6 (6,7.75)	0.738
Postoperative complications, n (%)			0.263
No Yes	28 (100.0%) 0 (0)	37 (92.5%) 3 (7.5%)	

### Subgroup analysis results

In the subgroup with a R.E.N.A.L. score of less than 7 points, there was no statistically significant difference in preoperative clinical data between the MR group and the control group of patients who underwent RAPN, indicating comparability (refer to **Table 3**). The operation time for the MR group was 15 min shorter than that of the control group (p< 0.05). Additionally, warm ischemia time and estimated intraoperative blood loss were reduced compared to those of the control group, although the statistical difference was not significant. No significant variances were observed in postoperative hospitalization time, drainage tube removal time, and other relevant intraoperative and postoperative indicators (refer to **Table 4**). There were no postoperative complications in the MR group. All resection margins were negative. In the control group, one postoperative complication was postoperative urinary fistula, and the resection margins were all negative.

**Table 3:** Comparison of preoperative general information (R.E.N.A.L. score less than 7).

			(%), $\bar{\text{x}}$ ± S, M (Q_1_, Q_3_)
	MR group (n = 22)	Control group (n = 27)	P
Age, mean ± sd (year)	57.36 ± 13.61	57.67 ± 11.50	0.933
Gender, n (%)			0.856
Male Female	16 (72.7%) 6 (27.3%)	19 (70.4%) 8 (29.6%)	
Height, mean ± sd (cm)	165.95 ± 8.55	164.52 ± 8.10	0.550
Weight, mean ± sd (kg)	67.55 ± 2.769	64.0 (55.00, 70.00)	0.219
BMI, mean ± sd (kg/m^2^)	24.45 ± 4.04	23.60 ± 3.00	0.401
R.E.N.A.L. score (Q_1_, Q_3_)	6 (5, 6)	5 (5, 6)	0.433
Tumor side, n (%)			0.238
Left Right	11 (50.0%) 11 (50.0%)	18 (66.7%) 9 (33.3%)	
Renal artery variation			0.990
No Yes	13 (59.1%) 9 (40.9%)	16 (59.3%) 11 (40.7%)	
Preoperative hemoglobin, mean ± sd (g/L)	142.91 ± 14.57	137.26 ± 13.79	0.171
Preoperative serum creatinine (Q_1_, Q_3_) (μmol/L)	78.55 ± 23.25	83.41 ± 26.90	0.507
ASA			0.493
1 2 3	1 (4.5%) 17 (77.3%) 4 (18.2%)	4 (14.8%) 19 (70.4%) 4 (14.8%)	

**Table 4:** Comparison of intraoperative and postoperative (R.E.N.A.L, score less than 7).

			(%), $\bar{\text{x}}$ ± S, M (Q_1_, Q_3_)
	MR group (n = 22)	Control group (n = 27)	P
Operation time, mean ± sd (min)	134.55 ± 23.45	150.19 ± 23.64	0.045
Warm ischemia time, mean ± sd (min)	20.14 ± 4.27	21.37 ± 5.11	0.370
Estimated amount of bleeding (Q_1_, Q_3_) (mL)	85 (50, 100)	90 (60, 110)	0.120
Postoperative hemoglobin, mean ± sd (g/L)	124.50 ± 14.51	119.19 ± 15.10	0.219
Postoperative serum creatinine (Q_1_, Q_3_) (µmol/L)	87.00 (66.25, 111)	93.00 (71.00, 116)	0.608
Hemoglobin changes (Q_1_, Q_3_) (g/L)	18.41 ± 8.59	18.07 ± 12.41	0.915
Changes in serum creatinine (Q_1_, Q_3_) (µmol/L)	12.5 (1.5, 15.5)	7.0 (3. 0, 15.0)	0.888
Postoperative hospital stay (Q_1_, Q_3_) (day)	8.09 ± 1.50	8.37 ± 2.31	0.627
Postoperative extubation time (Q_1_, Q_3_) (day)	6 (5, 7)	6 (6, 7)	0.901
Postoperative complications, n (%)			0.263
No Yes	22 (100%) 0 (0)	26 (96.3%) 1 (3.7%)	

In the subgroup with a R.E.N.A.L. score of not less than 7, there was no statistically significant difference in preoperative clinical data between the MR group and the control group, and they were comparable (refer to **Table 5** for details). In patients with complex renal tumors, the MR group exhibited significantly reduced operation time, warm ischemia time, and estimated intraoperative blood transfusion volume compared to the control group (p< 0.05). However, the variances in other related indicators did not show statistical significance (refer to **Table 6**). There were no postoperative complications in the MR group. All resection margins were negative. In the control group, there were two cases of postoperative complications, including one case of postoperative urinary fistula and one case of collecting system injury. All resection margins were negative.

**Table 5:** Comparison of preoperative general information (R.E.N.A.L. score of not less than 7).

			(%), $\bar{\text{x}}$ ± S, M (Q_1_, Q_3_)
	MR group (n = 6)	Control group (n = 13)	P
Age, mean ± sd (year)	48.56 ± 17.26	52.29 ± 16.54	0.669
Gender, n (%)			0.101
Male Female	3 (50.0%) 3 (50.0%)	11 (84.6%) 2 (15.4%)	
Height, mean ± sd (cm)	161.83 ± 9.11	168.31 ± 6.33	0.088
Weight, mean ± sd (kg)	61.50 ± 8.71	69.85 ± 13.48	0.186
BMI, mean ± sd (kg/m^2^)	23.42 ± 1.89	24.60 ± 4.12	0.512
R.E.N.A.L. score (Q_1_, Q_3_)	7.5 (7, 8.5)	8 (7, 8)	0.831
Tumor side, n (%)			1.000
Left Right	2 (33.3%) 4 (66.7%)	5 (38.5%) 8 (61.5%)	
Renal artery variation	3 (50.0%)	6 (46.2%)	1.000
No Yes	3 (50.0%)	7 (53.8%)	
Preoperative hemoglobin, mean ± sd (g/L)	133.83 ± 18.37	144.15 ± 14.58	0.203
Preoperative serum creatinine (Q_1_, Q_3_) (μmol/L)	61.50 (52.75, 187.75)	79 (64, 86)	0.368
ASA			0.710
1 2 3	0 (0) 6 (100.0%) 0 (0)	1 (7.7%) 10 (76.9%) 2 (15.4%)	

**Table 6:** Comparison of intraoperative and postoperative data (R.E.N.A.L. score of not less than 7).

			(%), $\bar{\text{x}}$ ± S, M (Q_1_, Q_3_)
	MR group (n = 6)	Control group (n = 13)	P
Operation time, mean ± sd (min)	140.83 ± 25.18	195.77 ± 23.08	<0.01
Warm ischemia time, mean ± sd (min)	21.17 ± 2.71	28.85 ± 7.57	0.029
Estimated amount of bleeding (Q_1_, Q_3_) (mL)	53.33 ± 5.164	109.23 ± 21.65	0.024
Postoperative hemoglobin, mean ± sd (g/L)	113.17 ± 18.69	120.69 ± 19.97	0.741
Postoperative serum creatinine (Q_1_, Q_3_) (µmol/L)	83.5 (59.25, 206.75)	88.00 (75.00, 115.00)	0.765
Hemoglobin changes (Q_1_, Q_3_) (g/L)	17.00 (5.75, 32.75)	22.00 (13.00, 27.50)	0.467
Changes in serum creatinine (Q_1_, Q_3_) (µmol/L)	14.50 (5.50, 24.25)	10.00 (6.50, 16.50)	0.639
Postoperative hospital stay (Q_1_, Q_3_) (day)	10.17 ± 2.99	10.31 ± 4.01	0.940
Postoperative extubation time (Q_1_, Q_3_) (day)	7.5 (5.75, 9.25)	7 (6, 11.50)	0.701
Postoperative complications, n (%)			0.566
No Yes	6 (100.0%) 0 (0)	11 (84.6%) 2 (15.4%)	

## Discussion

Renal cell carcinoma, also known as renal cell carcinoma or renal adenocarcinoma, ranks third among male urinary tract tumors and second among female urinary tract tumors. It is the most malignant tumor among urinary tract tumors (16). Partial nephrectomy has become the standard treatment for T1 stage renal cancer. Its main advantage lies in its ability to preserve renal function to a greater extent while ensuring the same level of efficacy, particularly in cases of bilateral renal tumors or anatomical and functional changes in patients with a solitary kidney (17, 18). RAPN was first documented in 2004. Over the years, it has been shown to outperform traditional laparoscopy by reducing bleeding time, conserving more renal tissue, and ultimately enhancing effectiveness. The procedure offers significant benefits in protecting postoperative renal function and boasts a shorter learning curve (19, 20). Nevertheless, ensuring maximal tumor integrity (i.e., negative resection margin) during robot-assisted partial nephrectomy, especially for complex and entirely endogenous renal tumors, remains a challenge. Minimizing perioperative and surgical costs, reducing complications, and optimizing renal function preservation continue to pose challenges for surgeons.

Although medical imaging has advanced rapidly in recent years, CT and MRI are able to detect kidney tumors at earlier stages. However, conventional imaging techniques such as CT and MRI still have their limitations. The visualization of tumors may not be clear, especially in terms of delineating tumor boundaries and understanding the spatial relationship between tumors near the renal hilum and the collecting system. Interpreting these images effectively requires significant experience, a keen eye for 2D and 3D anatomical details, among other skills (21). Inadequate preoperative planning and errors during renal pedicle exposure can increase the risk of vascular injury, potentially leading to severe bleeding that is challenging to manage or necessitating a switch to open surgery (22, 23). Consequently, there is a pressing need for improved imaging technology among surgeons to aid in preoperative assessments, surgical planning, and intraoperative navigation.

The new generation of MR technology combines the virtual world with real-time experiences, bridging the gap between virtual and physical realities. By seamlessly integrating virtual scenes into the real world and enabling interactions between the virtual environment, the physical world, and the user, MR technology revolutionizes the way that we perceive and interact with our surroundings. Verhey et al. suggest that MR technology can enhance preoperative surgical planning by providing a comprehensive 360° display of renal tumors without any blind spots. This immersive visualization aids in improving the efficiency of preoperative preparations and in enhancing the understanding of the patient’s anatomy (24). Liu et al. demonstrated the utility of MR in real-time imaging during laparoscopic partial nephrectomy. Their research highlighted how MR facilitates tumor tracking and localization, leading to enhanced precision in renal tumor resection (25). Furthermore, a retrospective study conducted by Yang et al. revealed that MR-assisted surgery can reduce intraoperative complications and enhance perioperative outcomes (26). These findings suggest that MR technology could serve as a valuable preoperative tool for planning complex renal tumor surgeries.

**Figure 6:**
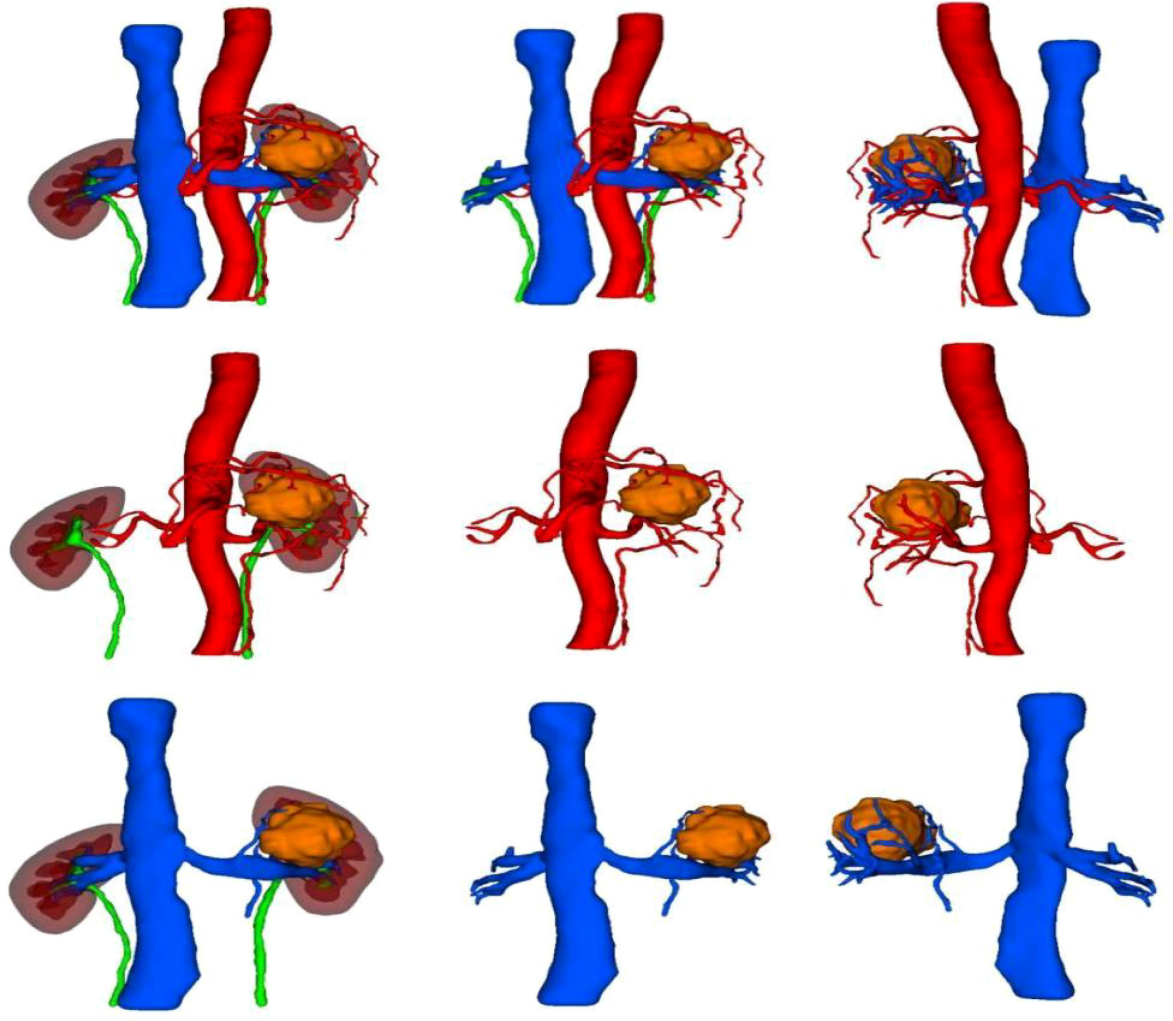
The blood vessels, including the arterial and venous systems surrounding the kidney and tumor, can be clearly displayed in various positions.

During the preoperative planning process, we observed that MR technology proves highly effective in visualizing variations in renal arteries. It offers clear insights into renal blood supply, including the presence of accessory arteries, and the status of branches, such as premature branches, thereby minimizing errors in interpretation of imaging data (see **Figure 6** for detail). This, in turn, reduces the risk of incomplete intraoperative blockade, which could lead to excessive blood loss or necessitate a conversion to open surgery. Furthermore, the technology’s ability to visualize the blood supply of renal artery branches enables selective blockade or even open surgery, facilitating superselective blocking when required. During the procedure, we utilize HoloLens glasses to project reconstructed images onto the operating table. By leveraging anatomical landmarks such as the liver, spine, and aorta, we seamlessly integrate virtual images with the patient’s actual anatomy, thereby enhancing our ability to pinpoint the tumor’s location, arterial blood supply, collecting system, and ureter. This streamlined approach significantly reduces the time required for intraoperative exploration of the renal artery and tumor. In certain scenarios, only local perirenal fat dissection is necessary. By overlaying the MR image onto the robot monitor image during surgery, we gain real-time insights into the relationship between the tumor and blood vessels without altering the field of view (refer to **Figure 4**) (27, 28). This feature is particularly advantageous for completely endophytic renal tumors. When combined with intraoperative ultrasound, the technology aids in rapid and precise tumor localization, guides renal parenchyma resection, and minimizes damage to critical blood vessels (29).

This study conducted a comprehensive analysis on patients to evaluate the benefits of MR in surgical procedures. The results revealed a notable decrease in operation time for the MR group compared to that for the control group (135.89 ± 23.494 min vs. 165.00 ± 34.32 min, P< 0.001), as well as a reduction in ischemia time (20.36 ± 3.956 min vs. 23.80 ± 6.889 min, P = 0.02). The software used in MR is capable of converting medical imaging data (such as CT and MRI) into detailed anatomical models. It employs 3D visualization techniques to render these models, effectively reproducing the unique 3D structure of individual anatomy. This is achieved through various methods, including transparency, rotation, zoom, and color adjustments, which facilitate the observation and understanding of the spatial relationships among organs and tissues. However, the accuracy of these models is limited by the quality of the input data, and processing large datasets requires significant computing power. For surgeons experienced in image recognition and processing, the learning curve is relatively short, but adequate training and support remain essential. By incorporating actual practice during surgery, surgeons could efficiently locate tumors, arteries, and differentiate the ureter. Moreover, preoperative planning with MR images enabled the development of detailed and rational tumor resection and reconstruction strategies, minimizing the need for impromptu intraoperative decisions. These factors collectively contributed to reduced operation time and warm ischemia time. Postoperative complications were notably lower in the MR group, with no significant issues among the 28 patients, in contrast to three cases in the control group—two urinary fistula cases (resolved with conservative observation) and one collecting system injury (managed with an indwelling ureteral stent). Although the difference was not statistically significant, a larger sample size may be necessary for further validation. Stratification based on R.E.N.A.L. score revealed that, in low-complexity renal tumor surgeries, MR technology primarily impacted operation time (134.55 ± 150.19 min vs. 150.19 ± 28.638 min, P = 0.045). Conversely, in complex renal tumor surgeries, the MR group exhibited reduced operation time (140.83 ± 25.183 min vs. 195.77 ± 23.080 min, P< 0.001) and warm ischemia time (21.17 ± 2.714 min vs. 28.85 ± 7.570 min, P = 0.029). Additionally, the MR group experienced significantly lower bleeding volume (53.33 ± 5.164 mL vs. 114.62 ± 80.376 mL, P = 0.018). The study highlighted the efficacy of MR in preoperative planning and intraoperative navigation for complex renal tumors, enhancing surgeons’ spatial awareness and reducing the need for impromptu decisions. This technology holds promise in improving surgical outcomes and streamlining the management of intricate renal tumors’ procedures (30). In addition, we will conduct follow-up statistics on progression-free survival, recurrence, and complications occurring after discharge in both the MR technology group and the control group. We will evaluate the diagnostic and therapeutic outcomes in the MR technology group to determine whether standardized diagnostic and treatment protocols for the new MR technologies should be implemented in the management of RAPN.

This study has several limitations. Firstly, it is a retrospective study, and, despite our efforts to use strict inclusion and exclusion criteria, there may still be selection bias. Secondly, the sample size is relatively small. Lastly, the model was developed using data from a single center. In the future, we will conduct larger-scale, multicenter prospective studies to validate our findings.

## Conclusion

In comparison to conventional RAPN, MR technology offers the potential to decrease operation time and warm ischemia time while maintaining similar tumor outcomes. Additionally, it enhances preoperative discussions, doctor-patient interactions, preoperative planning, and intraoperative navigation. Particularly beneficial for complex renal tumors, MR technology demonstrates notable advantages in this context.

## Data Availability

The data analyzed in this study is subject to the following licenses/restrictions: The data that support the findings of this study are not openly available due to reasons of sensitivity and are available from the corresponding author upon reasonable request. Requests to access these datasets should be directed to taozeng40709@sina.com.

## References

[1] Tempo J Logan C O’Callaghan M Kahokehr A Kichenadasse G D'Onise K. Bladder, penile, renal pelvis and testis cancers: A population based analysis of incidence and survival 1977-2013. Cancer Epidemiol. (2020) 65:101692. doi: 10.1016/j.canep.2020.101692 32151978

[2] Padala SA Barsouk A Thandra KC Saginala K Mohammed A Vakiti A. Epidemiology of renal cell carcinoma. World J Oncol. (2020) 11:79–87. doi: 10.14740/wjon1279 32494314 PMC7239575

[3] Cirillo L Innocenti S Becherucci F. Global epidemiology of kidney cancer. Nephrol Dial Transplant. (2024) 39:920–8. doi: 10.1093/ndt/gfae036 38341277

[4] Yuan L Liu P Zhao Z Wei Z Liu L Sun J. Cross-sectional survey on cigarette smoking in Chinese high-income areas. BMJ Open. (2022) 12:e056209. doi: 10.1136/bmjopen-2021-056209 PMC9058778 35487748

[5] Boussios S Devo P Goodall ICA Sirlantzis K Ghose A Shinde SD. Exosomes in the diagnosis and treatment of renal cell cancer. Int J Mol Sci. (2023) 24:14356. doi: 10.3390/ijms241814356 37762660 PMC10531522

[6] Campbell SC Clark PE Chang SS Karam JA Souter L Uzzo RG. Renal mass and localized renal cancer: evaluation, management, and follow-up: AUA guideline: part I. J Urol. (2021) 206:199–208. doi: 10.1097/JU.0000000000001911 34115547

[7] Macek P Cathelineau X Barbe YP Sanchez-Salas R Rodriguez AR. Robotic-assisted partial nephrectomy: techniques to improve clinical outcomes. Curr Urol Rep. (2021) 22:51. doi: 10.1007/s11934-021-01068-4 34622373

[8] Vartolomei MD Remzi M Fajkovic H Shariat SF. Robot-assisted partial nephrectomy mid-term oncologic outcomes: A systematic review. J Clin Med. (2022) 11:6165. doi: 10.3390/jcm11206165 36294486 PMC9605111

[9] Lv Z Chen G Chen X Li Y Bao E Hu K. Open versus robot-assisted partial nephrectomy for highly complex renal masses: a meta-analysis of perioperitive and functional outcomes. J Robot Surg. (2023) 17:1955–65. doi: 10.1007/s11701-023-01652-5 37415066

[10] Dubeux VT Zanier JFC Gabrich PN Carrerette FB Milfont JCA Damião R. Practical evaluation of the R. E. N. A. L. score system in 150 laparoscopic nephron sparing surgeries. Int Braz J Urol. (2022) 48:110–9. doi: 10.1590/S1677-5538 PMC8691232 34528773

[11] Hu C Sun J Zhang Z Zhang H Zhou Q Xu J. Parallel comparison of R.E.N.A.L, PADUA, and C-index scoring systems in predicting outcomes after partial nephrectomy: A systematic review and meta-analysis. Cancer Med. (2021) 10:5062–77. doi: 10.1002/cam4.4047 PMC8335816 34258874

[12] Dahlkamp L Haeuser L Winnekendonk G von Bodman C Frey UH Epplen R. Interdisciplinary comparison of PADUA and R.E.N.A.L scoring systems for prediction of conversion to nephrectomy in patients with renal mass scheduled for nephron sparing surgery. J Urol. (2019) 202:890–8. doi: 10.1097/JU.0000000000000361 31145034

[13] Butz B Jussen A Rafi A Lux G Gerken J. A taxonomy for augmented and mixed reality applications to support physical exercises in medical rehabilitation-A literature review. Healthcare(Basel). (2022) 10:646. doi: 10.3390/healthcare10040646 35455824 PMC9028587

[14] Checcucci E Amparore D Pecoraro A Peretti D Aimar R DE Cillis S. 3D mixed reality holograms for preoperative surgical planning of nephron-sparing surgery: evaluation of surgeons’ perception. Minerva Urol Nephrol. (2021) 73:367–75. doi: 10.23736/S2724-6051.19.03610-5 31486325

[15] Li G Dong J Wang J Cao D Zhang X Cao Z. The clinical application value of mixed-reality-assisted surgical navigation for laparoscopic nephrectomy. Cancer Med. (2020) 9:5480–9. doi: 10.1002/cam4.3189 PMC7402835 32543025

[16] Bukavina L Bensalah K Bray F Carlo M Challacombe B Karam JA. Epidemiology of renal cell carcinoma: 2022 update. Eur Urol. (2022) 82:529–42. doi: 10.1016/j.eururo.2022.08.019 36100483

[17] Pandolfo SD Cerrato C Wu Z Franco A Del Giudice F Sciarra A. A systematic review of robot-assisted partial nephrectomy outcomes for advanced indications: Large tumors(cT2-T3), solitary kidney, completely endophytic, hilar, recurrent, and multiple renal tumors. Asian J Urol. (2023) 10:390–406. doi: 10.1016/j.ajur.2023.06.001 38024426 PMC10659988

[18] Tan JS Sathianathen N Cumberbatch M Dasgupta P Mottrie A Abaza R. Outcomes in robot-assisted partial nephrectomy for imperative vs elective indications. BJU Int. (2021) 128Suppl3:30–5. doi: 10.1111/bju.15581 34448346

[19] Calpin GG Ryan FR McHugh FT McGuire BB. Comparing the outcomes of open, laparoscopic and robot-assisted partial nephrectomy: a network meta-analysis. BJU Int. (2023) 132:353–64. doi: 10.1111/bju.16093 37259476

[20] Pandolfo SD Wu Z Campi R Bertolo R Amparore D Mari A. Outcomes and techniques of robotic-assisted partial nephrectomy(RAPN) for renal hilar masses: A comprehensive systematic review. Cancers(Basel). (2024) 16:693. doi: 10.3390/cancers16040693 38398084 PMC10886610

[21] Talanki VR Peng Q Shamir SB Baete SH Duong TQ Wake N. Three-dimensional printed anatomic models derived from magnetic resonance imaging data: current state and image acquisition recommendations for appropriate clinical scenarios. J Magn Reson Imaging. (2022) 55:1060–81. doi: 10.1002/jmri.27744 PMC10326907 34046959

[22] Grosso AA Lambertini L Di Maida F Gallo ML Mari A Minervini A. Three-dimensional reconstruction and intraoperative ultrasonography: Crucial tools to safely approach highly complex renal masses. Int Braz J Urol. (2022) 48:996–7. doi: 10.1590/S1677-5538 PMC9747021 35594329

[23] Lin WC Chang CH Chang YH Lin CH. Three-dimensional Reconstruction of Renal Vascular Tumor Anatomy to facilitate accurate preoperative planning of partial nephrectomy. Biomedicine(Taipei). (2020) 10:36–41. doi: 10.37796/2211-8039.1078 PMC7735978 33854933

[24] Verhey JT Haglin JM Verhey EM Hartigan DE. Virtual, augmented, and mixed reality applications in orthopedic surgery. Int J Med Robot. (2020) 16:e2067. doi: 10.1002/rcs.2067 31867864

[25] Liu W Wang Y Wang Z Cao Z Yu Y Wang J. The application of mixed reality navigation system in laparoscopic partial nephrectomy for highly complex renal tumors (RENAL score ≥ 10): a retrospective cohort study. Int J Surg. (2024). doi: 10.1097/JS9.0000000000001983 PMC11745591 39093872

[26] Yang Y Gao Y Zhang XY Wang BJ Zhu J Zhang X. Mixed reality: A step further for planning complex renal tumors (RENAL nephrometry score of 7 or higher). J Endourol. (2022) 36:1136–42. doi: 10.1089/end.2021.0798 35262373

[27] Xin S Chen J Dongming L Wei X Yiran H. Application of three-dimensional reconstruction of renal tumor vessels to guide laparoscopic partial nephrectomy of hilar tumors and non-hilar tumors under zero ischemia. Asian J Surg. (2024) 47:216–21. doi: 10.1016/j.asjsur.2023.07.077 37574367

[28] Checcucci E Piana A Volpi G Quarà A De Cillis S Piramide F. Visual extended reality tools in image-guided surgery in urology: a systematic review. Eur J Nucl Med Mol Imaging. (2024) 51:3109–34. doi: 10.1007/s00259-024-06699-6 38589511

[29] Sun Y Wang W Zhang Q Zhao X Xu L Guo H. Intraoperative ultrasound: technique and clinical experience in robotic-assisted renal partial nephrectomy for endophytic renal tumors. Int Urol Nephrol. (2021) 53:455–63. doi: 10.1007/s11255-020-02664-y 33006090

[30] Porpiglia F Checcucci E Amparore D Piramide F Volpi G Granato S. Three-dimensional augmented reality robot-assisted partial nephrectomy in case of complex tumours (PADUA ≥10): A new intraoperative tool overcoming the ultrasound guidance. Eur Urol. (2020) 78:229–38. doi: 10.1016/j.eururo.2019.11.024 31898992

